# Comparison of Calcium and Barium Microcapsules as Scaffolds in the Development of Artificial Dermal Papillae

**DOI:** 10.1155/2016/9128535

**Published:** 2016-03-30

**Authors:** Yang Liu, Changmin Lin, Yang Zeng, Haihong Li, Bozhi Cai, Keng Huang, Yanping Yuan, Yu Li

**Affiliations:** ^1^Department of Histology and Embryology, Shantou University Medical College, Shantou, Guangdong 515000, China; ^2^Department of Burn and Plastic Surgery, The Second Affiliated Hospital, Shantou University Medical College, Shantou, Guangdong 515000, China; ^3^Tissue Engineering Laboratory, The First Affiliated Hospital, Shantou University Medical College, Shantou, Guangdong 515000, China; ^4^Department of Emergency, The Second Affiliated Hospital, Shantou University Medical College, Shantou, Guangdong 515000, China

## Abstract

This study aimed to develop and evaluate barium and calcium microcapsules as candidates for scaffolding in artificial dermal papilla. Dermal papilla cells (DPCs) were isolated and cultured by one-step collagenase treatment. The DPC-Ba and DPC-Ca microcapsules were prepared by using a specially designed, high-voltage, electric-field droplet generator. Selected microcapsules were assessed for long-term inductive properties with xenotransplantation into Sprague-Dawley rat ears. Both barium and calcium microcapsules maintained xenogenic dermal papilla cells in an immunoisolated environment and induced the formation of hair follicle structures. Calcium microcapsules showed better biocompatibility, permeability, and cell viability in comparison with barium microcapsules. Before 18 weeks, calcium microcapsules gathered together, with no substantial immune response. After 32 weeks, some microcapsules were near inflammatory cells and wrapped with fiber. A few large hair follicles were found. Control samples showed no marked changes at the implantation site. Barium microcapsules were superior to calcium microcapsules in structural and mechanical stability. The cells encapsulated in hydrogel barium microcapsules exhibited higher short-term viability. This study established a model to culture DPCs in 3D culture conditions. Barium microcapsules may be useful in short-term transplantation study. Calcium microcapsules may provide an effective scaffold for the development of artificial dermal papilla.

## 1. Introduction

Hair loss is most pervasive among middle-aged to elderly white men. By the age of 30 years, approximately 30% of white men are affected by hair loss; by the age of 50 and 70 years, at least 50 and 80% are affected, respectively [1, 2]. Current therapeutic management of baldness is via medication (which is not effective) or hair transplantation (which merely redistributes, rather than increasing the existing hairs). Therefore, novel and effective methods to treat hair loss are required.

The dermal papilla (DP) is situated at the base of the hair follicle (HF) and thought to be an important inducer of new HFs. HF formation occurs only once in a lifetime. In previous studies, researchers implanted DP beneath the upper half of amputated vibrissa HFs, then transplanted the DP into a follicular skin, and induced* de novo* hair growth [3–6]. Cultured dermal papilla cells (DPCs) could reconstitute new functioning DP* in vivo*. Although transplantation of DP could be a promising treatment option for baldness, the lack of DP with inductive properties is still a major obstacle.

Multilayer aggregated DPCs are essential for HF morphogenesis* in vivo* [7, 8]. Regeneration of HF can be achieved by transplanting dense multicellular aggregates or microtissues of DPCs [9].

For sophisticated development of tissues and growth of artificial organs, we must have materials with characteristics that closely emulate nature. In a previous study, we developed the concept of “artificial DP” by enclosing DPCs in an alginate-polylysine-alginate (APA) semipermeable membrane, which was then xenotransplanted into mouse ears and Sprague-Dawley rat footpads to induce HF formation and/or visible hair growth [10, 11]. However, the APA microcapsule is hollow and, when fragmented, the inside cells are exposed to immune cells. Also, studies on the long-term transplantation of artificial DP are lacking.

Animal studies and clinical trials have shown that alginate based products can provide long-term stability, biocompatibility, and viability of transplanted cells [12]. Barium (Ba) is commonly used to increase the mechanical stability. Even low molar concentrations of Ba in the gel solution can substantially decrease capsule swelling [13]. The other divalent cation usually used is calcium (Ca), which is not associated without cytotoxicity [14].

To determine a good scaffold material for the development of artificial DP, this study investigated the use of Ba and Ca microcapsules.

## 2. Material and Methods

### 2.1. Animals

The BALB/c mice and Sprague-Dawley rats, 3 to 6 months old and equal number of males and females, were obtained from the Center of Research Animals, Shantou University Medical College. Animals were maintained at the experimental animal center (SPF animal lab), Shantou University Medical College. All experiments were approved by the Ethics Committee on Research Animal Care at Shantou University Medical College (number SUMC2012092).

### 2.2. Cultivation and DPC Microcapsules

Normal human scalp specimens were obtained from selected patients with face-lift surgeries. DPCs were isolated and cultured as previously described [15]. The DPC-Ba and DPC-Ca microcapsules were prepared by using a specially designed, high-voltage, electric-field droplet generator (designed by the Institute of Cryobiological Engineering, Shanghai University of Science and Technology). In step 1, the cultured DPCs (5 × 10^5^ cells/mL) were suspended in a mixture of 2% (w/v) sodium alginate (Sigma), and then spherical droplets were formed by using the high-voltage electric droplet generator with optimized parameters. In step 2, after washing with 0.9% saline, the droplets were suspended in 1% (w/v) poly-L-lysine (MW 15000–22000, Sigma) solution for 10 min and transferred to 0.2% (w/v) sodium alginate solution for 4 min. It was then treated with 0.05 M sodium citrate for 6 min to liquefy the interior gel of the capsules. Ba microcapsules were prepared as in step 1 and washed with 0.9% saline. Thereafter, microcapsules were cultured with Dulbecco Modified Eagle Medium (DMEM) at 37°C. The concentrations of Ba and Ca required in the microcapsules were 11 g/L and 5.2 g/L of calcium chloride and barium chloride, respectively.

Microcapsule size was measured under an inverted microscope by using the micrometer. After 2 weeks of cultivation, microcapsules were stained with hematoxylin and eosin for routine histology. The viability of the microencapsulated DPCs was determined by 3-(4,5-dimethylthiazol-2yl)-2,5-diphenyl tetrazolium bromide (MTT) assay and Trypan blue staining by the routine process.

### 2.3. TEM Analysis of the Ultrastructure of Ba and Ca DPC Microcapsules

DPC-Ba and DPC-Ca microcapsules were prefixed with 2.5% glutaraldehyde for 2 hours at 1 to 4°C and washed with 0.1 mol/L phosphate-buffered saline. This mixture was then treated with 1% OsO4 solution for 2 hours and washed with 0.1 mol/L phosphate-buffered saline. Samples were dehydrated with alcohol and treated with PolyBed 812 resin overnight. Then, they were stained with uranyl acetate and lead citrate and examined by transmission electron microscopy (TEM) with a JEM1400 microscope (JEOL, Japan).

### 2.4. Analysis of Structural Stability of Microcapsules

About 1,000 each of DPC-Ba and DPC-Ca microcapsules were transferred into beakers and placed on a magnetic force whisk, which was run at 500 r/min. At this speed, the whisk was rotating at high speed, and the microcapsules knocked on the walls of the beaker. Structural stability was assessed by determining change in structural integrity at 10 and 60 min, using a CX31-12 inverted microscope (Olympus, Japan). Then, 1 mL each of Ba and Ca microcapsules of 400 *μ*m diameter were injected through the 7#, 9#, and 16# pinheads with maximum hand forces. The integrated microcapsules were counted using a CK2 phase-contrast microscope (Olympus, Japan), and the structural stability of microcapsules was evaluated.

### 2.5. Permeability of Artificial and Natural Membranes

Different diameter microcapsules were prepared by adjusting the parameters of the high-voltage electric-field droplet generator. The Ba and Ca microcapsules were divided into 3 groups based on size (*n* = 10 each): small (200 to 399 *μ*m), medium (400 to 599 *μ*m), and large (600 to 1000 *μ*m). These microcapsules were incubated with 3 molecular weights of FITC-dextran (1 mg/mL, 10, 40, and 70 kDa, resp., Invitrogen). Fluorescence intensities in microcapsules were measured at various time intervals by confocal microscopy (ACAS/Ultima312, Meridian Instruments, USA).

For detailed structure analysis, Ba and Ca microcapsules (*n* = 10), 400 *μ*m in diameter, and freshly isolated DP were incubated with 40 kDa FITC-dextran at 37°C, at various time intervals (10, 20, 30, 60, and 120 min, resp.). The fluorescence inside the microcapsules and DP cells was measured from the center and scanned every 5 *μ*m for 5 layers by confocal microscopy. The fluorescence signal was analyzed using the Image Analyze software. Each time point was assessed 10 times.

### 2.6. Biocompatibility Study of Microcapsules

The same number of DPC-Ba and DPC-Ca microcapsules was introduced into the peritoneal cavity of BALB/c mice (*n* = 8). Mice were sacrificed at 2 and 4 weeks, and the microcapsules were retrieved and observed under an inverted microscope.

### 2.7. Analysis of Hair Follicle Generation by DPC-Ba and DPC-Ca Microcapsules in Mice Model

Sprague-Dawley rats (*n* = 42) were anesthetized with ketamine, and the implantation sites were swabbed with 75% ethanol and 0.9% saline. About 1 mL of air was injected into the subcutaneous tissue of the hairless regions of rat ears to separate the epidermis and dermis [11]. A 2 mm incision was made in the right and left ears by using the tip of a scalpel blade. Approximately 0.1 mL of Ba or Ca microcapsules were implanted into the incision on the left ear by using a 16# injection needle. The incision was closed with the biological glue. Empty microcapsules were implanted into the right ear and were used as the control. Rats with both Ba and Ca microcapsules (*n* = 12 each) were sacrificed at 1, 2, 3, and 4 weeks. Rats with only Ca microcapsules (*n* = 18) were sacrificed at 12, 16, 20, 24, 28, and 36 weeks. The implantation sites were biopsied for histological evaluation.

### 2.8. Histology

Specimens were treated with 4% paraformaldehyde, dehydrated through a graded series of ethanol, washed with xylene, and embedded in paraffin wax. These treated specimens were cut into 4 *μ*m sections and stained with hematoxylin and eosin for routine histological evaluation.

### 2.9. Statistical Analysis

Data are presented as mean ± SD. Analysis of structural stability and biocompatibility of the microcapsules were evaluated by 2-tail Student's *t*-test. A *P* value < 0.05 was considered statistically significant.

## 3. Results

### 3.1. DPC-Ba and DPC-Ca Microcapsule Structure and Viability of Embedded Cells

**Figure 1:**
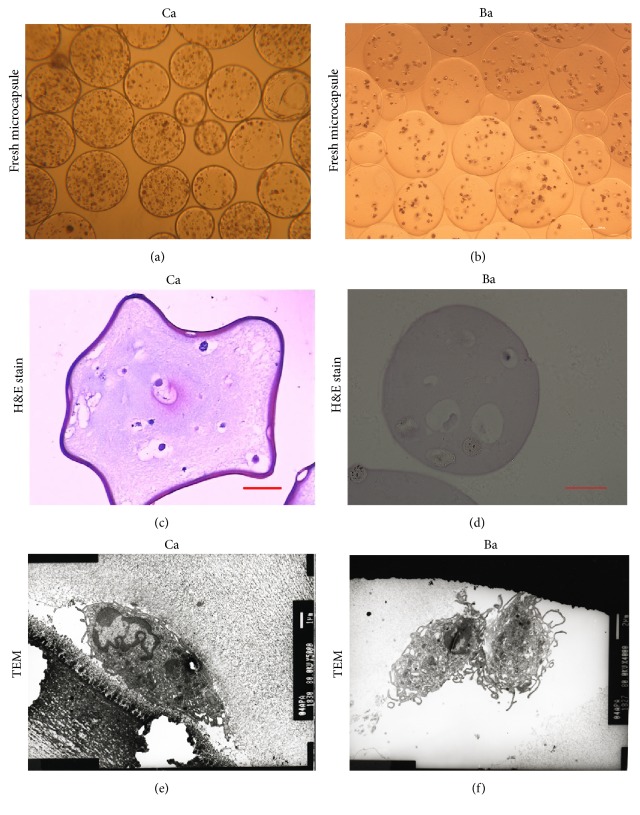
Ca and Ba DPC microcapsule structure. Microcapsule structures were accessed with light microcopy (a, b). The packaged DP cells and extracellular structures were further studied with H&E staining (c, d). Transmission electron microscopy was utilized to observed detail structures of microcapsule membrane and inside DP cells (red circles) (e, f). Ca (a) and Ba (b) microcapsules appeared round, smooth, and transparent under inverted microscopy. H&E staining showed extracellular matrix secreted around cells (red arrow) (c). Ba microcapsules showed scattered cells at all times, with no extracellular matrix (d). TEM confirmed that DPCs adhered to the Ca microcapsule membrane in 24 to 72 hours, and the cells retained their fine structure (e). The membrane of the Ba microcapsules was smooth, with solid and homogeneous core and cells inside showed many microvilli (red arrows) (f). (Bar = 100 *μ*m (a, b, c, and d), Bar = 1 *μ*m (e, f).)

Microscopy showed that Ba and Ca microcapsules were round, with smooth and transparent structure (Figures 1(a) and 1(b)). By adjusting the parameters of the high-voltage electric-field droplet generator to 7.0 kV voltage, injection speed to 55 mm/hour, and a distance of 10 mm, the diameter of the DPC microcapsules formed was maintained uniform at 0.4 mm. The DPCs embedded within the Ca microcapsules were either isolated or grouped in small clusters. At 12-hour culture, Ca microcapsules showed multilayered aggregates. For Ba microcapsules, the number of DPCs was constant, and the cells were either isolated or grouped as small clusters. At 24 hours after embedding, viability of microencapsulated cells by Trypan blue staining was 84.4% for Ca microcapsules and 75.4% for Ba microcapsules (*P* > 0.05).

Histology of Ca microcapsules revealed gradual aggregation of DPCs. Concurrently, cells showed secretion of extracellular matrix by hematoxylin and eosin staining (Figures 1(c) and 1(d)), which indicated continuous production of high amounts of glycosaminoglycan, a characteristic feature of the* in situ* conditions during anagen [2, 16]. For Ba microcapsules, cells were separate at all times, with no extracellular matrix production.

The TEM analysis revealed that the external microcapsule membrane and DPCs were surrounded by a fibrillar matrix consisting mainly of alginate (Figure 1(e)). TEM analysis also confirmed that the DPCs adhered to the microcapsule membrane for 24 to 72 hours and the cells retained their fine structure for 6–8 weeks under conventional culture conditions. The membrane of the Ba microcapsules was smooth, with homogeneous and solid contents inside the microcapsules. The DPCs were separate and rounded with the solid alginate (Figure 1(f)).

**Figure 2:**
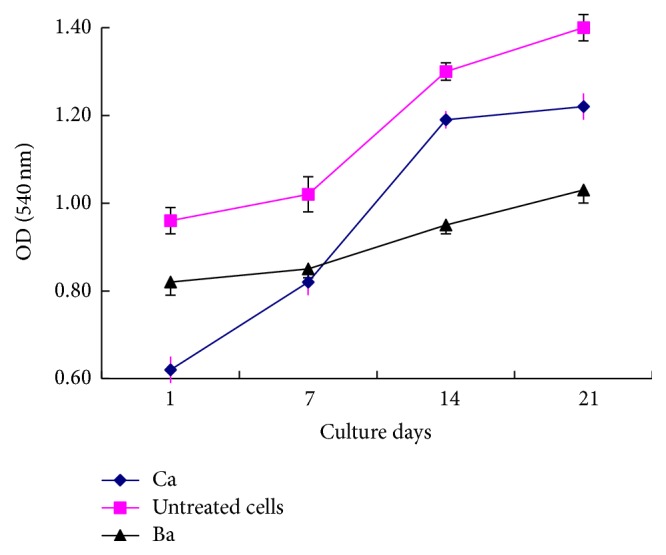
Viability and growth of embedded cells. Populations of alive cell in microencapsulated cells and untreated cells were determined by MTT assay. Data are represented as mean ± SD from 3 experiments.

At 48 hours, the viability of DPCs microencapsulated in Ba and Ca microcapsules was 90.5% and 85.3%, respectively. The MTT assay showed that the density of Ba and Ca microencapsulated DPCs was initially lower compared with the nonencapsulated DPCs but increased gradually (Figure 2). By day 14, the density of DPCs in Ca microcapsules was close to that of the control group.

### 3.2. Structural Stability of Ba and Ca Microcapsulated DPCs

**Figure 3:**
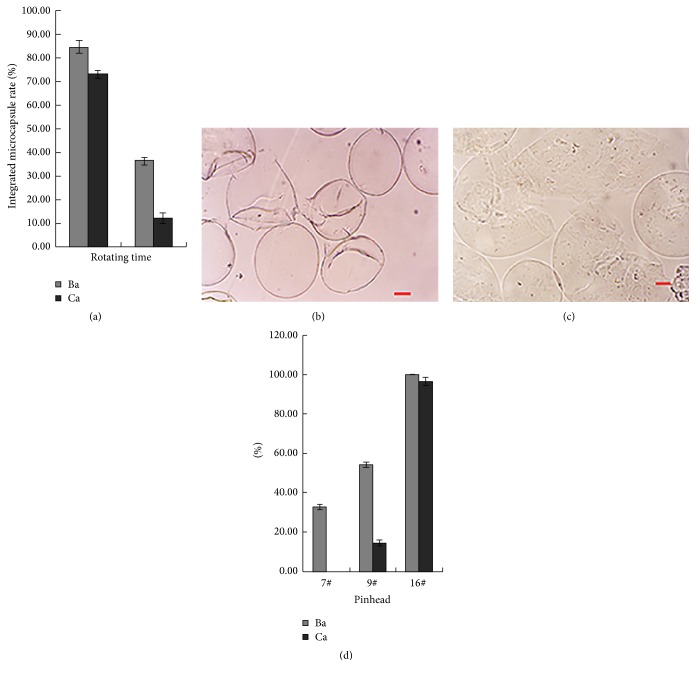
Structural stability microcapsules. Thousands each of DPC-Ba and DPC-Ca microcapsules were assessed by determining change in structural integrity under an inverted microscope at 10 and 60 min after being stirred in flask (a). Cells inside the fragmented Ca microcapsules escaped (b). Cells were retained inside the membrane of Ba microcapsules, even after fragmentation (c). 1 mL of Ba and Ca microcapsules of 400 *μ*m diameter were injected through 7#, 9#, and 16# pinheads, and integrated microcapsules were counted under a phase-contrast microscope (d). (Bar = 100 *μ*m.)

The structural stability was significantly higher for Ba compared with Ca microcapsules during whisking and injection. The numbers of Ba and Ca microcapsules that retained structural integrity at 10 min were 84.8% and 73.2%, respectively (*P* < 0.05). At 60 min, their numbers were 36.3% and 12.0%, respectively (*P* < 0.01) (Figure 3(a)). The contents inside the Ca microcapsules escaped when fragmented (Figure 3(b)). However, the cells were retained within the membrane for Ba microcapsules, even after fragmentation (Figure 3(c)).

The percentages of microcapsules that remained intact following the injection of 400 *μ*m Ba microcapsules through the 7#, 9#, and 16# pinheads were 32.8%, 54.1%, and 100%, respectively, and those for Ca microcapsules were 0%, 14.6%, and 96.5%, respectively (Figure 3(d)).

### 3.3. Permeability of Ba and Ca Microcapsule Membranes

**Figure 4:**
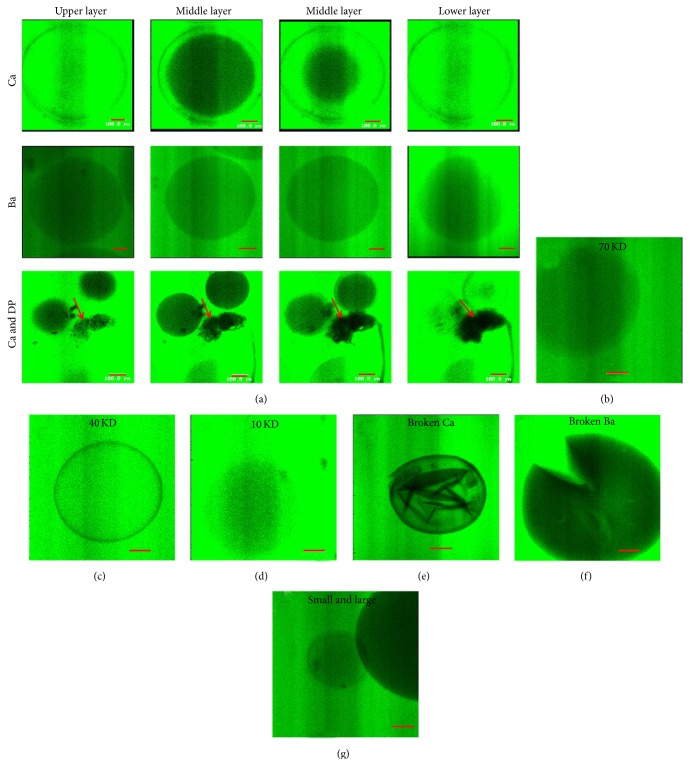
Permeability of artificial and natural membranes. (a) The diffusion of fluorescence in Ca and Ba microcapsules and fresh DP (red arrow) was monitored by scanning from the center every 5 *μ*m ×4 times by confocal microscopy after being permeabilized for 60 min. (b, c, d) The Ca microcapsules permeabilized for 30 minutes with different molecular weight of FITC-dextran. The medium and FITC-dextran entered the broken Ca capsules immediately (e) but not into Ba microcapsules even after fragmentation (f). (g) The fluorescence was stronger for 200 *μ*m microcapsules compared with 600 *μ*m diameter Ca microcapsules. (Bar = 100 *μ*m.)

To test the permeability of microcapsules to nutrient materials, the microcapsules were treated with FITC-dextran. Confocal microscopy of Ca microcapsules from top to bottom revealed homogenously diffused fluorescence gradually coalescing in sharp concentric circles into the center of microcapsules. Specifically, because of the liquefied alginate core of Ca microcapsules, the fluorescence could diffuse into the core of Ca microcapsules within 60 min (Figure 4(a)). However, the Ba and fresh DP microcapsules both showed significantly lower fluorescence compared to the Ca microcapsules (*P* < 0.01), with no sharp concentric circles (Figure 4(a)). The Ba and fresh DP microcapsules were not completely filled with fluorescence in 120 min. The fluorescence intensity inside Ca microcapsules was related to the molecular weight of FITC-dextran (10 > 40 > 70 kDa) (Figures 4(b), 4(c), and 4(d)). When Ca microcapsules were fragmented, medium and FITC-dextran leaked into microcapsules immediately (Figure 4(e)). However, no FITC-dextran was detected inside the solid medium of Ba microcapsules, even after Ba microcapsules were fragmented (Figure 4(f)). The fluorescence was stronger for 200 *μ*m compared with 600 *μ*m diameter Ca microcapsules (Figure 4(g)).

### 3.4. Biocompatibility of DPC-Ba and DPC-Ca Microcapsules

**Figure 5:**
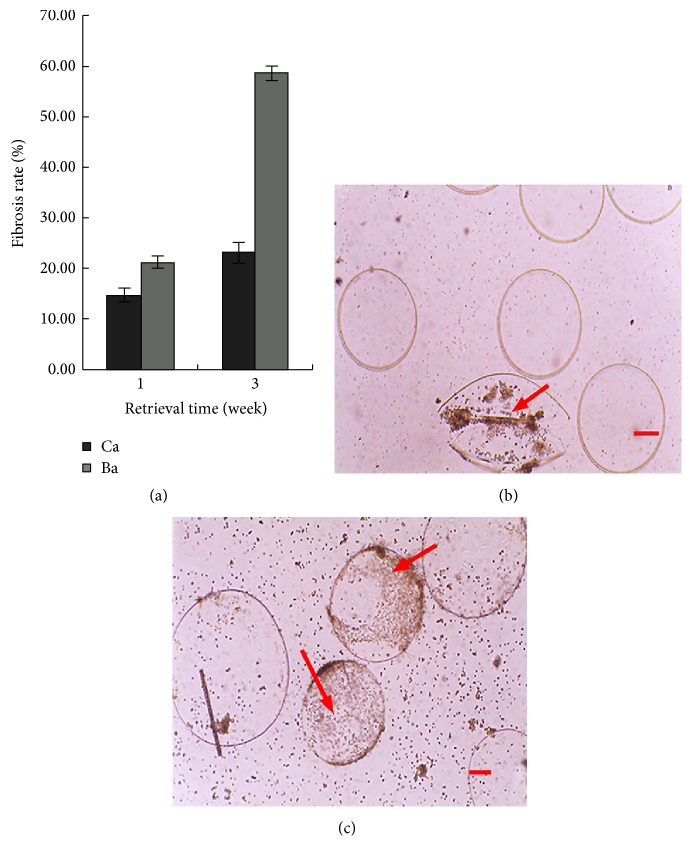
Biocompatibility of microcapsules. The same number of empty DPC-Ba and DPC-Ca microcapsules was introduced into the peritoneal cavity of mice and retrieved after 1 and 3 weeks for measurement of rate of fibrosis (a). One week after transplantation into the mouse peritoneal cavity, the retrieved Ca (b) and Ba (c) microcapsules showed increased fibrosis (2.3% and 5.9% (*P* < 0.01), resp.), with surrounding inflammatory cells. (Bar = 100 *μ*m.)

One week after transplantation into the peritoneal cavity, the retrieval rates of Ba and Ca microcapsules were 84.3% and 78.9%, respectively (*P* > 0.05) (data not shown). After 3 weeks, the retrieval rates were 76.8% and 70.2%, respectively (*P* > 0.05). After 1 week, the rates of fibrosis for Ba and Ca microcapsules were 1.47% and 2.10% (*P* > 0.05), respectively. After 3 weeks, the rates of fibrosis were 2.31% and 5.85%, respectively (*P* < 0.01, Figure 5(a)). When the microcapsules were fragmented, the cells within the Ca microcapsules escaped (Figure 5(b)) while those within the Ba microcapsules were retained in microcapsule membranes.

### 3.5. Functional Study of DPC Microcapsules in Rat Ears

#### 3.5.1. Short-Term Study (First 12 Weeks)

**Figure 6:**
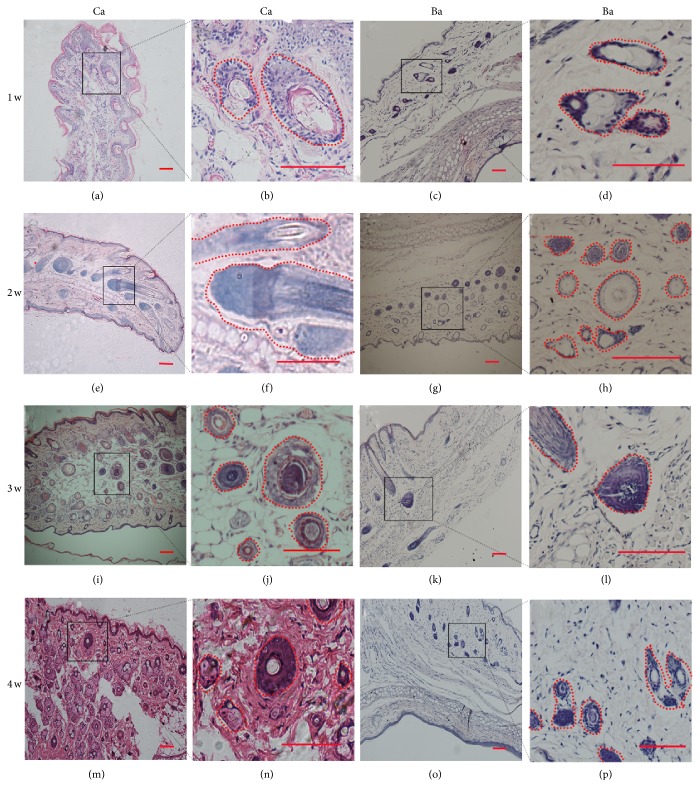
Short-term study of DPC-Ca and DPC-Ba microcapsules transplanted into rat ears. Large hair follicles formed after Ca microcapsule implantation at weeks 1–4 (a, b, e, f, i, j, m, and n). At 2 weeks, large DP formed near the transplanted site without the surrounding DPC microcapsule (e, f). No abnormal hair follicle structures were found in Ba microcapsules at 1 to 2 weeks after transplantation (c, d, g, and h). At 3 to 4 weeks, Ba (k, l, o, and p) microcapsules produced large hair follicles but fewer compared with Ca (i, j, m, and n) microcapsules. (Bar = 100 *μ*m.)

When implanted into hairless regions of SD rat ears, both DPC-Ba and DPC-Ca microcapsules induced the* de novo* growth of a large number of HFs in rat ears within 2 to 4 weeks. When compared with Ba microcapsules, Ca microcapsules showed stronger induction. The induced HF number, size, and visible fibers were significantly greater with Ca microcapsules compared with Ba microcapsules (Table 1). The number and size of abnormal HF and sebaceous glands were greater with Ca microcapsules compared with the Ba microcapsules (Figure 6). The new large HFs in case of Ca microcapsules showed the basic morphological features of human HFs and visible hair fibers at 6 weeks. The longest fibers grew to 7 to 8 mm. These abnormal fibers remained for 3 to 4 weeks and then fell off. Also the large HFs in case of Ca microcapsules remained until 8 weeks and reduced gradually in number but were absent in Ba microcapsules within 2 to 3 weeks. While the inflammatory cells gathered and blood vessels proliferated at the injected sites during the first week following the transplantation for the Ba microcapsules, the Ca microcapsules did not produce these reactions until 24 weeks.

#### 3.5.2. Long-Term Study (12 to 36 Weeks)

**Figure 7:**
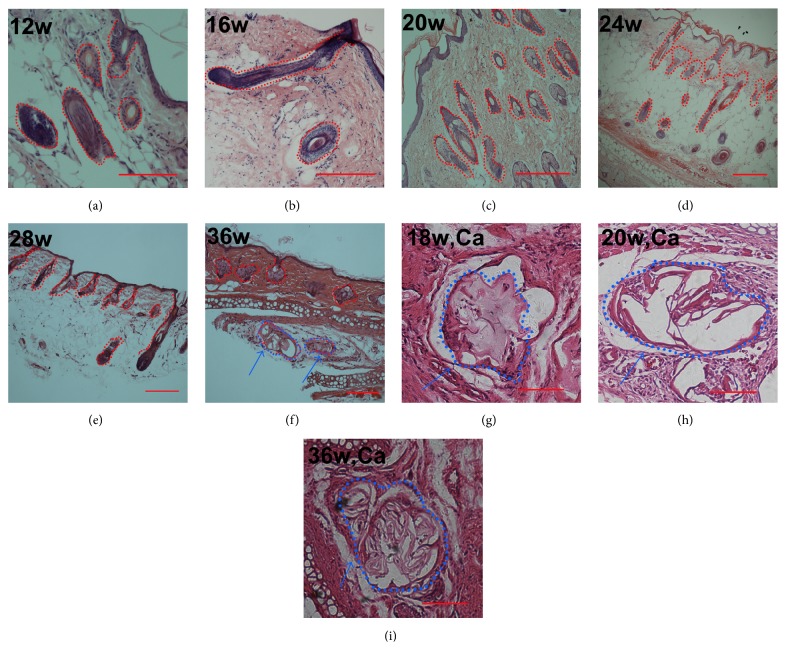
Long-term study of DPC-Ca microcapsules transplanted into rat ears and the histology of Ca microcapsules at different weeks. From 12 to 36 weeks, the number and size of large hair follicles decreased (a, b, c, d, e, and f). At 18 weeks, Ca microcapsules were found at the transplanted site, with no surrounding inflammatory cells (g). At 20 weeks, some inflammatory cells were around the microcapsules (h). At 36 weeks the number of inflammatory cells was reduced, with fibers around the microcapsules (i). (Bar = 100 *μ*m.) Red circle: microcapsules and formed HFs. Blue circle and arrow: microcapsules surrounded associated with inflammatory cells.

Interestingly, the DPC-Ca microcapsules showed some changes synchronously in the formation of HFs in rat ears. From 12 to 20 weeks, the number of large HF structures was reduced. From 20 to 32 weeks, only a few abnormal HFs remained (Figures 7(a), 7(b), and 7(c)). Before 20 weeks, only DPC microcapsules were found in the sections with no inflammatory cells around the microcapsules, indicating no serious immune response. From 20 to 36 weeks, the DPC microcapsules were associated with few inflammatory cells and small blood vessel proliferation (Figure 7(h)). After 36 weeks, some DPC microcapsules were surrounded by inflammatory cells and wrapped with fibers (Figure 7(i)). A few abnormal HFs were found in the section. Control samples showed no marked changes at the implantation site (data not shown).

## 4. Discussion

The DP from human scalp is oval, with an approximately 200 to 300 *μ*m long axis. Every DP has about 500 cells [16]. The proliferation rate of DPCs was high* in vitro*, which indicated that enough cells could be harvested for transplantation. Compared with other cells, DPCs have low metabolism for maintaining multilayer aggregation when cultured* in vitro* [5]. In our previous study, we found that DPCs provided some signals to induce HF reconstruction but not form the structures of new HFs [10, 11]. Here, we investigated the culture of DPCs in a 3D microenvironment using alginate microcapsules. Several studies have demonstrated the benefits of 3D culture for DP to maintain its HF induction property [10, 11, 17]. In the present study, we compared 2 different alginate microcapsules, the Ba microcapsules and Ca microcapsules.

Cell viability was better with DPC-Ca microcapsules compared with DPC-Ba microcapsules. The permeability was higher for Ca microcapsules compared with the Ba microcapsules. Also the largest molecule that could permeate through the APA membranes was <70 kDa for Ca microcapsules but was <40 kDa for Ba microcapsules. Ba and Ca microcapsules may have different core textures, liquid in case of Ca microcapsules but solid for Ba microcapsules. This may explain why the DPCs had better viability with Ca microcapsules compared with the Ba microcapsules. The permeability was greater for Ca microcapsules (200 to 400 *μ*m) compared with the fresh DP membranes, which suggested that Ca microcapsules can maintain DPC viability well. A higher “permeability” allows better passage of small molecules such as nutrients, oxygen, and growth factors and thereby helps keep the cells alive. Furthermore, Ca microcapsules but not Ba microcapsules secreted extracellular matrix from DPCs. Despite all cells surviving in both alginate microcapsules, cell viability in APA was better for Ca microcapsules compared with the Ba microcapsules in the long-term study. In another study [18], primary mammalian chondrocytes and intervertebral disc cells maintained higher viability and proliferation rate and produced higher sulfated glycosaminoglycan content when encapsulated in Ca microcapsules compared to the encapsulation in Ba alginate.

Biocompatibility was better for Ca microcapsules compared with Ba microcapsules in the long term following transplantation. Ba microcapsules have an elastic/gel like core, for better structural stability than Ca microcapsules. If Ca microcapsules are blocked, the inside cells would be immediately exposed to immune response. In transplanted animals, before 30 weeks, we observed no inflammatory cells in case of Ca microcapsules. Therefore, Ca microcapsules could contain and immunoprotect inner cells for a long time.

Although the compatibility between the Ba microcapsules and the receptor was less compared with that with Ca microcapsules, the Ba microcapsules showed more structural stability when compared with Ca microcapsules. Ba microcapsules may be beneficial in other fields such as short-term induced study or some special sites.

It is worth noticing that, in our biocompatibility and animal implantation assays, both Ba and Ca microcapsules showed resistance to* in vivo* degradation. These results might be due to large size and the stability of composing materials of microcapsules. In addition, the failure of transplanted animal to keep the hair follicle structure in the long term might also contribute to the stability of microcapsules, resulting in insufficient blood and nutrition supplies. Chemical modifications have been suggested to improve the* in vivo* degradation of transplant supporting materials [19]. Further related studies are needed to address those issues.

Some studies have shown other 3D culture systems. For instance, long-term 3D histoculture (supported by Gelfoam®) of whiskers isolated from transgenic mice was established, with nestin driving green fluorescent protein (ND-GFP), and stem cells were shown to traffic from the BA toward the DP area over a 2-week period, extensively growing out onto Gelfoam forming nerve-like structures [20]. Interestingly, the growing whisker sensory nerve was highly enriched in ND-GFP cells which affected its elongation as well as interaction with other nerves, indicating a major impact for the multipotent nestin-positive stem cells in the hair follicle on follicle sensory-nerve growth and regeneration [21, 22]. In addition, nestin-expressing multipotent stem cells were found in the fungiform papilla in the tongue; they showed typical markers of multiple cell types such as nerve cells, glial cells, and keratinocytes, indicating that nestin-expressing fungiform papilla cells and the nestin-expressing hair follicle stem cells have high similarity [23]. Finally, Gelfoam-histocultured whisker follicles were shown to produce growing pigmented and unpigmented hair shafts, suggesting that Gelfoam histoculture can support extensive hair-shaft growth as well as hair follicle sensory-nerve growth from isolated hair follicles for long periods of time. Therefore, Gelfoam histoculture of hair follicles allows prolonged evaluation of novel agents to promote hair growth [24]. It might be a good alternative 3D culture system choice.

The capability of DP cells to induce HF formation was suggested to be due to secreted factors such as cytokines [25]. Pluripotent stem cells expressing nestin have been identified in both bulge area and DP in hair follicle [26, 27]. The cells developed from bulge area have been proved to contain the pluripotency to different into other nonfollicle cells including neuron [27–32]. Indeed, the bulge area nestin-expressing stem cells seemed to be with better pluripotency than DP derived stem cells. The previous and present studies have tested the effect of DP cell transplantation in promoting hair follicle formation.

## 5. Conclusions

In conclusion, we have established a model to culture DPCs in 3D culture conditions. Both Ba and Ca microcapsules keep xenogenic DPCs in an immunoisolated environment and induce* de novo* HF formation before inflammatory cell aggregation. As a scaffold for DPCs, Ca microcapsule scaffolds were better compared with Ba scaffolds in terms of biocompatibility, permeability, and cell viability. Implanted DPCs may provide important materials to induce HF reconstruction. Compared with other 3D culturing methods [17, 31, 32], the DPC-Ca microcapsules may have a significant advantage in xenotransplantation. Also the implanted DP on Ca microcapsule scaffolds can induce new and native DP regeneration and is fiber-wrapped after long-term transplantation.

## Figures and Tables

**Table 1:** Numbers of observed hair follicles after microcapsule injection (in single visible field under microscope with 10x amplification).

Week	Ba	Ca
1	7.7 ± 1.5	13.3 ± 1.5
2	12.7 ± 0.6	15.7 ± 1.5
3	16.0 ± 1.0	23.0 ± 2.0
4	11.7 ± 1.5	20.0 ± 1.0
